# Low Serum Dehydroepiandrosterone Is Associated With Diabetic Kidney Disease in Men With Type 2 Diabetes Mellitus

**DOI:** 10.3389/fendo.2022.915494

**Published:** 2022-06-15

**Authors:** Xinxin Zhang, Jinfeng Xiao, Xin Li, Jingqiu Cui, Kunling Wang, Qing He, Ming Liu

**Affiliations:** ^1^ Department of Endocrinology and Metabolism, Tianjin Medical University General Hospital, Tianjin, China; ^2^ NHC Key Laboratory of Hormones and Development, Tianjin Medical University, Tianjin, China; ^3^ Tianjin Institute of Endocrinology, Tianjin, China

**Keywords:** dehydroepiandrosterone, dehydroepiandrosterone sulfate, diabetic kidney disease, type 2 diabetes mellitus, androgen

## Abstract

**Background:**

The associations of dehydroepiandrosterone (DHEA) and dehydroepiandrosterone sulfate (DHEAS) with diabetic kidney disease (DKD) remained unclear. Thus, this cross-sectional study aimed to explore the associations of DHEA and DHEAS with the risk of DKD in patients with T2DM.

**Methods:**

The information of 1251 patients with T2DM were included in this study. Serum DHEA and DHEAS were quantified using liquid chromatography-tandem mass spectrometry assays. Multivariate logistic regression analyses were used to assess the associations of DHEA and DHEAS with DKD as well as high urine albumin to creatinine ratio (ACR).

**Results:**

In men with T2DM, the risk of DKD decreased with an increasing DHEA concentration after adjustment for traditional risk factors; the fully adjusted OR (95% CI) for tertile3 vs tertile1 was 0.37 (0.19-0.70; P = 0.010 for trend). Similarly, when taking high ACR as the outcome, low DHEA levels were still significantly associated with increased odds of high ACR (OR, 0.37; 95% CI, 0.19–0.72 for tertile3 vs tertile1; P = 0.012 for trend). The restricted cubic spline showed that the risk of DKD gradually decreased with the increment of serum DHEA levels (P-overall = 0.007; P-nonlinear = 0.161). DHEAS was not independently associated with the risk of DKD in men. In contrast, no significant relationships were found between DHEA and DHEAS and the risk of DKD in women (all P > 0.05).

**Conclusions:**

In men with T2DM, low serum DHEA levels were independently related to the risk of DKD after adjustment for traditional risk factors. Our finding highlights the potential role of DHEA in the development of DKD in men with T2DM.

## Introducton

Diabetic kidney disease (DKD), defined by diabetes with albuminuria or reduced glomerular filtration rate, is one of the common microvascular complications of diabetes. DKD is a leading cause of end-stage renal disease, affecting 40% patients with type 2 diabetes mellitus (T2DM) (1). The prevalence of diabetes-related end-stage renal disease remains increasing despite the improvement in therapies and risk management (2). DKD has been proved to increase the risk of hyperuricemia and cardiovascular disease (CVD) (3, 4). Moreover, in Asian individuals, patients with DKD are at high risks of all-cause and CVD mortality (5).

Dehydroepiandrosterone (DHEA) and its sulfate ester (DHEAS), as precursors of androgen, are the most abundant sex hormones in the human circulation. Concentrations of DHEA and DHEAS gradually decrease with age in men and women (6). The DHEA secretion follows a circadian rhythm and influenced by adrenocorticotropic hormone and glucocorticoid. Additionally, the renal clearance of DHEAS is decreased when renal function is reduced. Evidence has shown that DHEA and DHEAS improve insulin sensitivity, inhibit vascular inflammation, increase endothelial cell proliferation, and reverse systemic vascular remodeling (7–10). Moreover, epidemiological studies in general population have reported that low DHEA and DHEAS concentrations are related to the risk of T2DM, coronary heart disease, and all-cause and cardiovascular mortality (11–14). In men with T2DM, low DHEAS has been shown to be associated with urinary albumin excretion (15). However, high DHEAS levels were proved to be related to decreased renal function evaluated by creatinine clearance in lean, young men (16). Meanwhile, the associations of DHEA and DHEAS with DKD remained unclear.

Accordingly, this cross-sectional study aimed to explore the associations of DHEA and DHEAS with the risk of DKD in patients with T2DM.

## Methods

### Study Population

**Figure 1:**
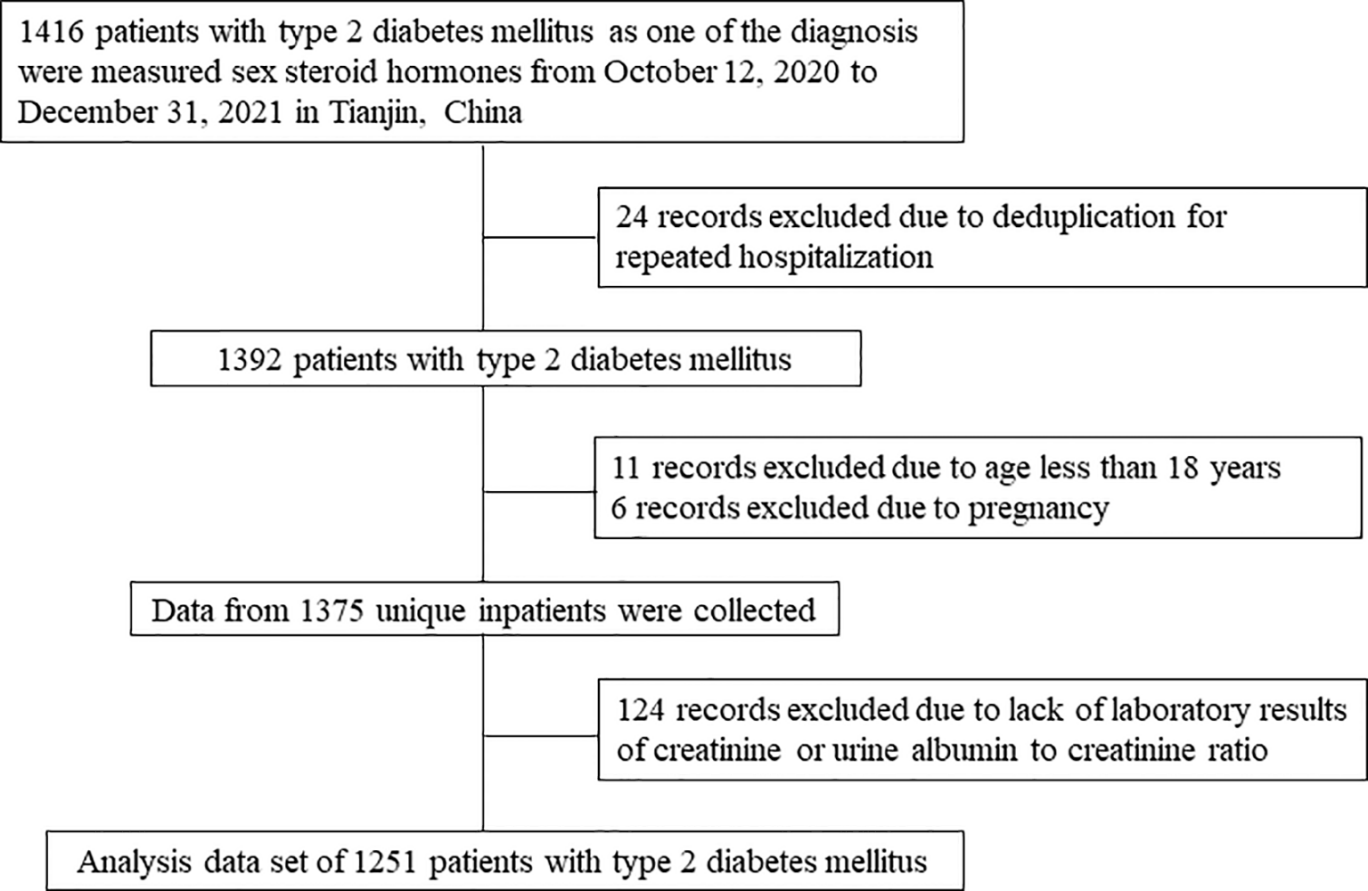
Flow chart of identification of study population. There were 1251 patients with T2DM included in the final analysis.

This cross-sectional study was conducted in the Department of Endocrinology and Metabolism, Tianjin Medical University General Hospital in Tianjin, China. There were 1416 patients with T2DM hospitalized and measured sex steroid hormones in our department from October 12, 2020 to Dec 31, 2021. Only one record was selected if there were multiple hospitalization records for one participant. Patients aged less than 18 years or pregnant were excluded. Patients without laboratory results of creatinine or urine albumin to creatinine ratio (ACR) were also excluded from the analysis. **Figure 1** shows the flow chart of identification of study population. Therefore, there were totally 1251 inpatients with T2DM included in this study.

This study received ethical approval from the institutional review board of Tianjin Medical University General Hospital and was carried out in accordance with the Helsinki Declaration in 1995 (as revised in Fortaleza, Brazil, October 2013). The requirement for informed consent was waived (approval number: IRB2020-YX-027-01) because the patients’ information was extracted from electronic medical records, and the patients’ identities were kept anonymous.

### Information Collection

The information about age, sex, insurance type, cigarette smoking, alcohol drinking, medical history, height, weight, systolic blood pressure (SBP), diastolic blood pressure (DBP), total cholesterol (TC), triglycerides (TG), high-density lipoprotein (HDL), low-density lipoprotein (LDL), fasting blood glucose (FBG), glycosylated hemoglobin (HbA1c), uric acid (UA), creatinine, ACR, and the use of renin angiotensin aldosterone system (RAAS) and sodium-glucose cotransporter 2 (SGLT2) inhibitors was collected from the electronic medical records.

Participants were divided into three groups according to types of medical insurance: patients with medical insurance for urban workers or non-working residents, or patients without any kind of medical insurance. Body mass index (BMI) was calculated by patients’ weight in kilogram divided by the square of whose height in meter. The estimate glomerular filtration rate (eGFR) was calculated based on the Chronic Kidney Disease Epidemiology Collaboration (CKD-EPI) equation (17).

### Measurements of DHEA and DHEAS

After admission to hospital, overnight fasting blood samples (at least 10 h of fasting) were drawn in the morning which were immediately sent to the Laboratory of Endocrinology and Metabolism at Tianjin Medical University General Hospital for the measurement of DHEA and DHEAS. Serum DHEA and DHEAS were quantified by using liquid chromatography-tandem mass spectrometry assays. After pretreatment, the samples were loaded to a Jasper™ HPLC system coupled to an AB SCIEX Triple Quad™ 4500MD mass spectrometer with a heated nebulizer ionization source in positive ion mode. There were six calibration standards and two quality control samples included in each set of samples. The calibration curves used linear regression with 1/x^2^ weighting, and the correlation coefficients were all greater than 0.99.

### Definitions

Diabetes was defined as FBG ≥7.0 mmol/L, 2-hour blood glucose ≥11.1 mmol/L, HbA1c ≥6.5%, self-reported history of diabetes, or use of hypoglycemic drugs (18). Hypertension was defined as SBP ≥140mmHg, DBP ≥90mmHg, self-reported history of hypertension, or use of blood-pressure-lowering drugs (19). Dyslipidemia was defined as TC ≥6.2 mmol/L, TG ≥2.3 mmol/L, LDL cholesterol ≥4.1 mmol/L, HDL cholesterol <1.0 mmol/L, or use of lipid-lowering drugs (20). High ACR was diagnosed as ACR>30 mg/g and DKD was diagnosed as ACR>30 mg/g or eGFR<60 mL/min/1.73 m^2^ (21).

### Statistical Analysis

Continuous variables were shown as mean ± standard deviation (normal distribution) or median with interquartile range (skewed distribution) and categorical variables were shown as numbers with percentages. Continuous variables were compared by using Student’s t-tests (normal distribution) or Mann-Whitney U tests (skewed distribution) and categorical variables by chi-squared tests. Multivariate logistic regression analyses were used to assess the associations of DHEA and DHEAS with DKD as well as high ACR, the results were shown as odds ratios (ORs) and 95% confidence intervals (CIs). Serum levels of DHEA and DHEAS were also equally divided into tertiles, with the lowest tertile taken as reference in the multivariate logistic regression analyses. The restricted cubic spline was used to assess the dose-response association of DHEA with DKD after adjusting for confounding factors. DHEA was normalized in the multivariate logistic regression analyses and restricted cubic spline. The knots were placed at the 5th, 35th, 65th, and 95th percentiles and the reference value was at 50th percentile of DHEA based on the distribution. A two-sided P value < 0.05 was considered statistically significant. SPSS for Windows (version 25.0, Chicago, IL, USA) and R software (version 4.1.3, R Foundation) were used for analyses.

## Results

### Clinical Characteristics of the Participants

**Figure 2:**
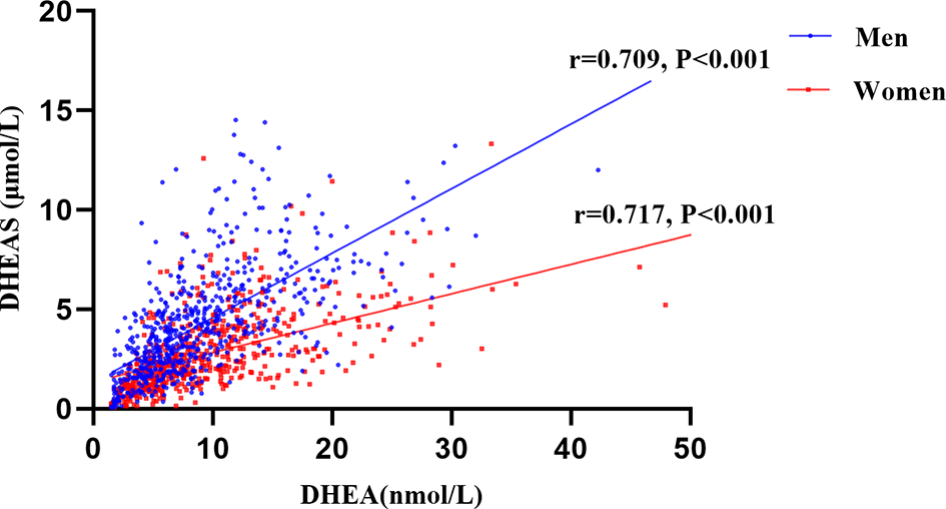
The correlations between serum dehydroepiandrosterone (DHEA) and dehydroepiandrosterone sulfate (DHEAS) among men and women in this study. Figure 2 indicates that serum DHEA and DHEAS were highly collinear in this study. (r = 0.709; P < 0.001 among men; r = 0.717; P < 0.001 among women).

**Table 1** shows the clinical characteristics of study population grouped according to gender and the presence of DKD. Men with DKD had higher mean age, duration of T2DM, SBP, UA, and prevalence of hypertension than those without DKD (all P < 0.001). Obviously, men with DKD had lower eGFR levels and higher ACR levels (all P < 0.001). Moreover, serum DHEA and DHEAS levels were statistically lower among men with DKD than those without DKD (all P < 0.001). In addition, the serum DHEA and DHEAS were highly collinear in this study (r = 0.709; P < 0.001 among men; r = 0.717; P < 0.001 among women, **Figure 2**).

**Table 1:** Characteristics of patients with and without DKD.

	Men(n=653)				Women(n=598)		
	Non-DKD	DKD	P		Non-DKD	DKD	P
Participants, %	434 (66.5)	219 (33.5)	–		382 (63.9)	216 (36.1)	–
Age, years	54.46 ± 13.77	59.36 ± 14.34	**<0.001**		56.94 ± 14.13	58.24 ± 16.03	0.319
BMI, Kg/m2	27.11 ± 5.03	27.50 ± 5.31	0.397		27.12 ± 6.11	28.13 ± 6.06	0.074
Current smoking, %	199 (46.3)	88 (40.9)	0.198		15 (4.0)	12 (5.7)	0.335
Current drinking, %	195 (45.3)	81 (37.5)	0.057		10 (2.6)	8 (3.8)	0.438
Insurance type, %			0.283				0.206
Urban workers	361 (83.2)	171 (78.1)			318 (83.2)	169 (78.2)	
Non-working urban residents	48 (11.1)	31 (14.2)			44 (11.5)	36 (16.7)	
Self-pay	25 (5.8)	17 (7.8)			20 (5.2)	11 (5.1)	
Duration of type 2 diabetes mellitus, year	6.00 (0.50,12.00)	10.00 (4.00,20.00)	**<0.001**		6.00 (1.00,13.00)	10.00 (2.00,19.00)	**<0.001**
Dyslipidemia, %	333 (77.3)	173 (80.8)	0.298		258 (68.1)	178 (82.4)	**<0.001**
Hypertension, %	232 (53.5)	161 (73.5)	**<0.001**		188 (49.2)	157 (72.7)	**<0.001**
Blood pressure, mmHg							
Systolic	133.21 ± 15.92	143.33 ± 20.53	**<0.001**		132.59 ± 16.48	140.80 ± 20.03	**<0.001**
Diastolic	83.74 ± 11.15	85.15 ± 13.28	0.177		79.82 ± 10.04	82.81 ± 13.04	**0.004**
Use of RAAS inhibitors, %	135 (31.2)	83 (38.2)	0.072		96 (25.2)	72 (33.5)	**0.031**
Use of SGLT-2 inhibitors, %	44 (10.4)	29 (13.7)	0.218		32 (8.5)	16 (2.7)	0.687
TC, mmol/L	4.84 ± 1.87	4.81 ± 1.34	0.776		5.05 ± 1.47	5.15 ± 1.30	0.411
TG, mmol/L	1.72 (1.21,2.49)	1.79 (1.23,2.66)	0.989		1.66 (1.25,2.31)	1.88 (1.36,2.78)	**0.001**
HDL-C, mmol/L	1.04 ± 0.26	1.06 ± 0.29	0.492		1.15 ± 0.28	1.09 ± 0.27	0.010
LDL-C, mmol/L	2.92 ± 0.94	2.90 ± 1.03	0.815		3.00 ± 1.02	3.05 ± 0.97	0.542
FBG, mmol/L	7.67 ± 2.92	7.56 ± 2.97	0.669		7.42 ± 2.63	8.37 ± 3.14	**<0.001**
HbA1c, %	8.65 ± 2.33	8.79 ± 2.17	0.458		8.21 ± 2.07	8.87 ± 1.94	**<0.001**
UA, μmol/L	354.82 ± 100.19	389.44 ± 121.52	**<0.001**		324.80 ± 93.94	367.46 ± 183.41	**0.022**
DHEA, nmol/L	8.66 (5.78,12.71)	6.17 (4.03,8.51)	**<0.001**		8.34 (5.05,12.30)	7.11 (4.27,12.12)	**0.027**
DHEAS, μmol/L	4.13 (2.59,6.02)	2.90 (1.76,4.77)	**<0.001**		2.33 (1.31,3.97)	2.17 (1.25,3.73)	0.453
eGFR, ml/(min*1.73m^2^)	112.88 ± 17.55	90.02 ± 31.85	**<0.001**		106.47 ± 17.65	94.48 ± 33.54	**<0.001**
ACR, mg/g	9.52 (5.70,15.25)	132.35 (46.84,465.40)	**<0.001**		11.00 (6.80,17.40)	80.40 (43.00,246.20)	**<0.001**

BMI body mass index; RAAS, renin angiotensin aldosterone system; SGLT2, sodium-glucose cotransporter 2; TC, total cholesterol; TG, triglycerides; HDL-C, high density lipoprotein cholesterol; LDL-C, low density lipoprotein cholesterol; FBG, fasting blood glucose; HbA1c, glycosylated hemoglobin; UA, uric acid; DHEA, dehydroepiandrosterone; DHEAS, dehydroepiandrosterone-sulfate; eGFR, estimated glomerular infiltration rate; ACR, albumin to creatinine ratio. Bold results are statistically significant.

Likewise, duration of T2DM, SBP, DBP, TG, FBG, HbA1c, and UA were higher in female patients with DKD than in those without DKD (all P < 0.05). Women with DKD had lower eGFR levels, higher ACR levels, and higher frequencies of hypertension, dyslipidemia, and use of RAAS inhibitors (all P < 0.05). The serum DHEA levels were lower in women with DKD than in those without DKD (P = 0.027, **Table 1**).

### Prevalence of DKD by Tertiles of DHEA and DHEAS

**Figure 3:**
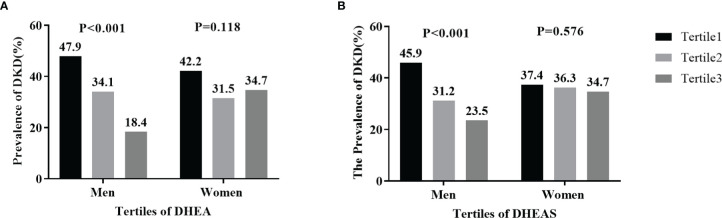
Prevalence of diabetic kidney disease (DKD) by tertiles of serum dehydroepiandrosterone (DHEA) and dehydroepiandrosterone sulfate (DHEAS) in men and women: **(A)** DHEA; **(B)** DHEAS. Figure 3 shows that the percentages of men with DKD significantly decreased in accordance with increasing tertiles of serum DHEA and DHEAS (both P < 0.001).

**Figure 3** displays the prevalence of DKD by sex and tertiles of DHEA and DHEAS. In men with T2DM, the prevalence of DKD gradually decreased in accordance with increasing tertiles of DHEA and DHEAS, with 47.9% and 45.9% in tertile1, 34.1% and 31.2% in tertile2, and 18.4% and 23.5% in tertile3, respectively (all P < 0.001). However, no statistically significant trends were found about the percentages of DKD in women with T2DM (all P > 0.05).

### Associations of DHEA and DHEAS With the Risk of DKD

The adjusted ORs for the associations of DHEA and DHEAS with DKD as well as high ACR in men are presented in **Table 2**. After adjustment for age, current smoking, current drinking, insurance type, BMI, duration of diabetes, dyslipidemia, SBP, DBP, FBG, HbA1c, UA, and use of RAAS and SGLT2 inhibitors, the risk of DKD decreased with an increasing DHEA concentration; the fully adjusted OR (95% CI) for tertile3 vs tertile1 was 0.37 (0.19-0.70; P = 0.010 for trend). Moreover, per standard deviation (SD) increment of DHEA was related to a 37% decrease in the risk of DKD among men (OR, 0.63; 95% CI, 0.46–0.87; P < 0.05). Similarly, when taking high ACR as the outcome, low DHEA levels were still significantly associated with increased odds of high ACR (OR, 0.37; 95% CI, 0.19–0.72 for tertile3 vs tertile1; P = 0.012 for trend). Low DHEAS levels were also statistically related to the risk of DKD and high ACR in the fully adjusted model (DKD: OR, 0.46; 95% CI, 0.24–0.87 for tertile3 vs tertile1; P = 0.023 for trend; high ACR: OR, 0.46; 95% CI, 0.24–0.87 for tertile3 vs tertile1; P = 0.011 for trend). However, when analyzed as a continuous variable, the associations of DHEAS with DKD or high ACR were not statistically significant (DKD and high ACR: all OR, 0.85; 95% CI, 0.65–1.12; all P > 0.05).

**Table 2:** Odds ratios of DKD among men by different status of DHEA and DHEAS.

	Odds ratios (95% CI)		
	Model 1	Model 2	Model 3
**DKD**			
DHEA, nmol/L			
Tertile1	Reference	Reference	Reference
Tertile2	**0.60 (0.40,0.89)**	**0.62 (0.41,0.93)**	0.66 (0.39,1.12)
Tertile3	**0.28 (0.17,0.46)**	**0.29 (0.18,0.49)**	**0.37 (0.19,0.70)**
P for trend	**<0.001**	**<0.001**	**0.010**
1SD increment of DHEA	**0.55 (0.43,0.70)**	**0.55 (0.43,0.71)**	**0.63 (0.46,0.87)**
DHEAS, μmol/L			
Tertile1	Reference	Reference	Reference
Tertile2	**0.59 (0.40,0.88)**	**0.60 (0.40,0.91)**	**0.53 (0.31,0.91)**
Tertile3	**0.46 (0.29,0.73)**	**0.49 (0.31,0.79)**	**0.46 (0.24,0.87)**
P for trend	**0.002**	**0.007**	**0.023**
1SD increment of DHEAS	0.83 (0.67,1.02)	0.85 (0.69,1.05)	0.85 (0.65,1.12)
**High ACR**			
DHEA, nmol/L			
Tertile1	Reference	Reference	Reference
Tertile2	0.68 (0.45,1.00)	0.70 (0.46,1.06)	0.74 (0.43,1.26)
Tertile3	**0.31 (0.19,0.50)**	**0.32 (0.19,0.54)**	**0.37 (0.19,0.72)**
P for trend	**<0.001**	**<0.001**	**0.012**
1SD increment of DHEA	**0.57 (0.44,0.74)**	**0.58 (0.45,0.75)**	**0.62 (0.45,0.86)**
DHEAS, μmol/L			
Tertile1	Reference	Reference	Reference
Tertile2	**0.58 (0.39,0.88)**	**0.60 (0.39,0.91)**	**0.47 (0.27,0.81)**
Tertile3	**0.48 (0.30,0.77)**	**0.52 (0.32,0.84)**	**0.46 (0.24,0.87)**
P for trend	**0.002**	**0.012**	**0.011**
1SD increment of DHEAS	0.84 (0.68,1.03)	0.87 (0.70,1.08)	0.85 (0.65,1.12)

Model 1: adjusts for age.

Model 2: model 1 + current smoking, current drinking, insurance type.

Model 3: model 2 + BMI, duration of diabetes, dyslipidemia, SBP, DBP, FBG, HbA1c, UA, and use of RAAS and SGLT2 inhibitors.

DKD, diabetic kidney disease; CI, confidence intervals; DHEA, dehydroepiandrosterone; DHEAS, dehydroepiandrosterone-sulfate; SD, standard deviation; ACR, albumin to creatinine ratio; BMI body mass index; SBP, systolic blood pressure; DBP, diastolic blood pressure; FBG, fasting blood glucose; HbA1c, glycosylated hemoglobin; UA, uric acid; RAAS, renin angiotensin aldosterone system**;** SGLT2, sodium-glucose cotransporter 2. Bold results are statistically significant.

In women with T2DM, serum DHEA levels were negatively associated with the odds of DKD in model 3 (OR, 0.86; 95% CI, 0.50–1.50; P = 0.045 for trend). However, this association was not statistically significant when taking DHEA as a continuous variable (OR, 0.95; 95% CI, 0.74–1.22; P > 0.05). In addition, no significant associations were found between DHEA and DHEAS and the risk of high ACR in women (all P >0.05, **Table 3**).

**Table 3:** Odds ratios of DKD among women by different status of DHEA and DHEAS.

	Odds ratios (95% CI)		
	Model 1	Model 2	Model 3
**DKD**			
DHEA, nmol/L			
Tertile1	Reference	Reference	Reference
Tertile2	**0.64 (0.42,0.97)**	**0.61 (0.40,0.93)**	**0.50 (0.29,0.88)**
Tertile3	0.77 (0.49,1.19)	0.79 (0.51,1.23)	0.86 (0.50,1.50)
P for trend	0.107	0.071	**0.045**
1SD increment of DHEA	0.82 (0.65,1.02)	0.82 (0.66,1.02)	0.95 (0.74,1.22)
DHEAS, μmol/L			
Tertile1	Reference	Reference	Reference
Tertile2	0.98 (0.65,1.48)	1.00 (0.66,1.52)	0.90 (0.53,1.53)
Tertile3	0.96 (0.62,1.50)	0.90 (0.57,1.42)	0.93 (0.52,1.65)
P for trend	0.986	0.875	0.922
1SD increment of DHEAS	1.01 (0.84,1.22)	0.99 (0.82,1.19)	1.02 (0.80,1.29)
**High ACR**			
DHEA, nmol/L			
Tertile1	Reference	Reference	Reference
Tertile2	0.66 (0.44,1.01)	**0.63 (0.41,0.97)**	0.58 (0.33,1.01)
Tertile3	0.77 (0.49,1.20)	0.79 (0.50,1.24)	0.90 (0.52,1.56)
P for trend	0.151	0.107	0.133
1SD increment of DHEA	0.82 (0.65,1.02)	0.82 (0.66,1.02)	0.94 (0.73,1.21)
DHEAS, μmol/L			
Tertile1	Reference	Reference	Reference
Tertile2	0.94 (0.62,1.42)	0.95 (0.63,1.45)	0.91 (0.54,1.55)
Tertile3	0.93 (0.59,1.45)	0.87 (0.55,1.37)	0.90 (0.51,1.60)
P for trend	0.932	0.827	0.923
1SD increment of DHEAS	1.00 (0.83,1.20)	0.98 (0.81,1.18)	1.00 (0.79,1.26)

Model 1: adjusts for age.

Model 2: model 1 + current smoking, current drinking, insurance type.

Model 3: model 2 + BMI, duration of diabetes, dyslipidemia, SBP, DBP, FBG, HbA1c, UA, and use of RAAS and SGLT2 inhibitors.

DKD, diabetic kidney disease; CI, confidence intervals; DHEA, dehydroepiandrosterone; DHEAS, dehydroepiandrosterone-sulfate; SD, standard deviation; ACR, albumin to creatinine ratio; BMI body mass index; SBP, systolic blood pressure; DBP, diastolic blood pressure; FBG, fasting blood glucose; HbA1c, glycosylated hemoglobin; UA, uric acid; RAAS, renin angiotensin aldosterone system; SGLT2, sodium-glucose cotransporter 2. Bold results are statistically significant.

**Figure 4:**
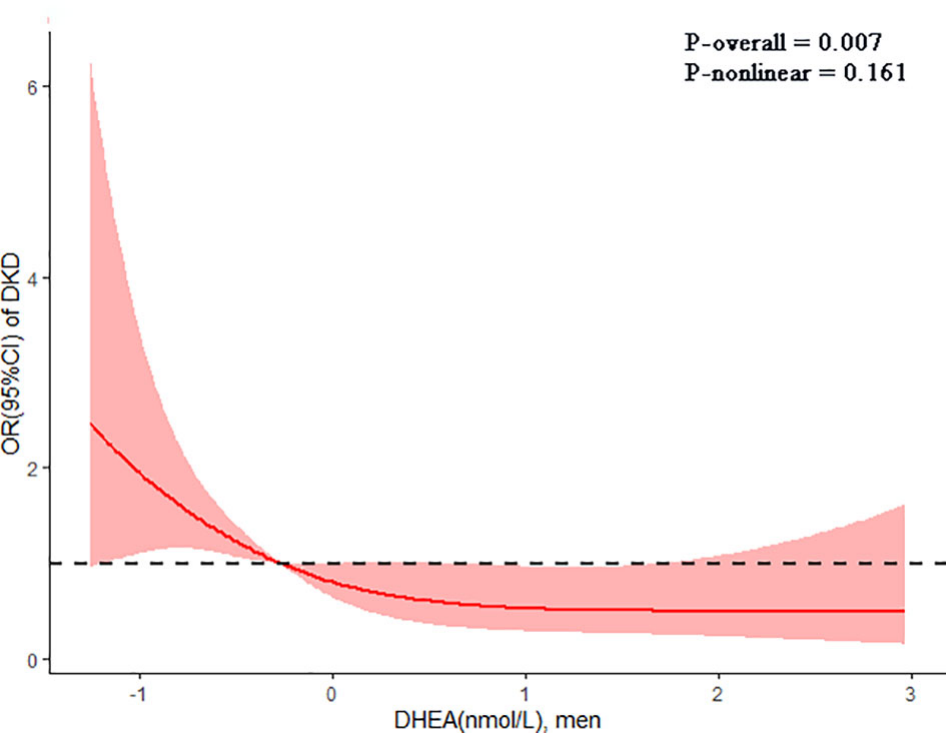
The dose-response association of dehydroepiandrosterone (DHEA) with diabetic kidney disease (DKD) in men shown by the restricted cubic spline. The line indicated the adjusted ORs for serum DHEA levels in men. 95% CI was shown by shaded areas. Adjust for age, current smoking, current drinking, insurance type, BMI, duration of diabetes, dyslipidemia, SBP, DBP, FBG, HbA1c, UA, and use of RAAS and SGLT2 inhibitors.

The restricted cubic spline reflected the dose-response association of DHEA with the risk of DKD in men (**Figure 4**). The risk of DKD gradually decreased with the increment of serum DHEA levels after adjusting for confounding factors (P-overall = 0.007; P-nonlinear = 0.161).

## Discussion

In this cross-sectional study, we provided the evidence about the associations of DHEA and DHEAS with the risk of DKD in patients with T2DM. Our results showed that, in men with T2DM, low serum DHEA levels were independently related to the risk of DKD after adjustment for traditional risk factors. In contrast, no significant relationships were found between DHEAS and the risk of DKD in men or women with T2DM.

DHEA has been proved to be beneficial in the progression of macrovascular diseases in previous studies. A prospective study including men aged 40-70 years has shown that low serum DHEA concentrations were related to incident ischemic heart disease, independent of traditional risk factors (22). In the Osteoporotic Fractures in Men study, serum DHEA has been found to predict the risk of developing coronary heart disease among elderly men (12). Additionally, a cross-sectional study enrolling patients with T2DM from Shanghai, China showed that low DHEA levels were associated with the presence of CVD in male patients (23). The links between DHEA and macrovascular diseases can be attributed to inhibition of vascular inflammation and platelet aggregation and reversion of vascular remodeling mediated by DHEA (8, 10, 24).

However, there were conflicting results about the relationships between DHEA and microvascular complications. A population-based, cross-sectional study including 5445 participants aged 50 years or more from the Rotterdam Study reported that DHEA was not statistically related to the risk of microvascular damage in men or women, evaluated by retinal arteriolar and venular calibers (25). However, another cross-sectional study enrolling patients with T2DM from Shanghai, China showed that postmenopausal women with higher DHEA levels were at increased odds of DKD (23). Unlike results in previous study, our data showed that low serum DHEA levels were independently related to the risk of DKD in men with T2DM after adjustment for traditional risk factors. Perhaps the differences in study design, participants included, and confounding factors adjusted could partly explain the conflicting conclusions. Further studies are needed to better clarify this issue.

Animal experiments indicated beneficial effect of DHEA on metabolism and renal injury despite conflicting results shown by epidemiological studies. In old female rats, DHEA treatment could change fatty acid profiles in serum and adipose tissue, decrease body weight and adiposity, and, thus, increase insulin sensitivity (26, 27). For dexamethasone-treated or diabetic rabbits, DHEA improved insulin sensitivity, lipid levels, directly inhibited renal gluconeogenesis, delayed the onset of diabetes, and alleviated renal oxidative stress and albuminuria (28–30). In hyperglycemic rats, DHEA treatment was also proved to prevent the oxidative damage in the kidneys (31, 32). Moreover, DHEA prevented lipid peroxidation and cell growth inhibition induced by high glucose concentrations in rat mesangial cells (33).

The evidences in animal experiments result in possible benefits of DHEA supplement in the prevention of chronic complications of diabetes. Some clinical trials in human showed the expected benefits in metabolism with DHEA treatment but others showed no significant metabolic changes. For women with adrenal insufficiency, 50 mg DHEA treatment for 3 months significantly improved insulin sensitivity (34). Additionally, in healthy men and women, DHEA therapy decreased insulin resistance, lipids, inflammatory cytokines, visceral fat, subcutaneous fat and, thus, had a potential impact in prevention of diabetes (35, 36). However, in a randomized, placebo-controlled, double-blind trial enrolling 112 healthy, elderly men and women, DHEA replacement could not improve insulin secretion or postprandial glucose metabolism pattern (37). The fat distribution, lipid profiles, or insulin action were also not changed in healthy men and women with DHEA replacement (38, 39). Even for patients with adrenal insufficiency, DHEA supplement for several months could not influence fasting glucose, insulin, lipids, or endothelial function (40, 41). Due to the small sample sizes of studies mentioned above, further clinical trials with large samples are still needed to confirm the effect of DHEA replacement on glucose metabolism and complications of diabetes.

The relationship between DHEAS and renal function was inconsistent evaluated by previous studies. A cross-sectional study recruiting 928 men (mean age: 18.5 ± 1.2) showed that DHEAS was inversely related to creatinine clearance in lean participants (16). The targeted metabolomics evaluating 450 plasma metabolites also proved that DHEAS was also inversely associated with eGFR in 616 adults (42). However, in patients with T2DM, low DHEAS concentrations were proved to be a risk predictor of urinary albumin excretion among men and women (43, 44). In addition, low DHEAS levels increased the risk of all-cause mortality in CKD hemodialysis men (45). Nevertheless, in adolescents with type 1 diabetes mellitus, DHEAS was not statistically associated with the risk of microalbuminuria after adjustment for cofounding risk factors (46). In this study, we did not find statistically significant associations of DHEAS with DKD as well as high ACR in men or women with T2DM. DHEAS, as the sulfation form of DHEA, is regarded as inactive metabolites because it cannot activate classical steroid receptors. Moreover, hydrophilic properties of the sulfation decrease the membrane permeation and movement of DHEAS from the circulation to peripheral tissues. Perhaps the inactive form and hydrophilic properties of sulfated steroid hormones could partly explain the different roles of DHEA and DHEAS in the presence of DKD. Future studies are needed to better clarify the mechanisms of this phenomenon.

This study had several limitations. First, the associations of DHEA and DHEAS with DKD did not indicate causation due to the cross-sectional design of the study; Second, we did not collect all factors related to DKD, such as eating habits, which might limit the multivariate analyses. Third, the sample size of our study was relatively small. Thus, future researches are needed to base on more large samples. Finally, this study recruited inpatients with T2DM; therefore, the conclusion could not represent the overall diabetic population.

In conclusion, in men with T2DM, low serum DHEA levels were independently related to the risk of DKD after adjustment for traditional risk factors. In contrast, no significant relationships were found between DHEAS and the risk of DKD in men or women. Our finding highlights the potential role of DHEA in the development of DKD in men with T2DM. Further prospective studies are needed to confirm the association in future.

## Data Availability Statement

The raw data supporting the conclusions of this article will be made available by the authors, without undue reservation.

## Ethics Statement

The studies involving human participants were reviewed and approved by the institutional review board of Tianjin Medical University General Hospital. Written informed consent for participation was not required for this study in accordance with the national legislation and the institutional requirements.

## Author Contributions

XZ, JX, and XL designed research, collected clinical data, and wrote the manuscript. JC was involved in clinical data collection and revision of the paper. ML, QH, and KW designed the study, revised and edited the manuscript paper. All authors contributed to the article and approved the submitted version.

## Funding

This work was supported by the National Natural Science Foundation of China (81830025 and 81620108004), the National Key R&D Program of China (2019YFA0802502), and Tianjin Key Medical Discipline (Specialty) Construction Project.

## Conflict of Interest

The authors declare that the research was conducted in the absence of any commercial or financial relationships that could be construed as a potential conflict of interest.

## Publisher’s Note

All claims expressed in this article are solely those of the authors and do not necessarily represent those of their affiliated organizations, or those of the publisher, the editors and the reviewers. Any product that may be evaluated in this article, or claim that may be made by its manufacturer, is not guaranteed or endorsed by the publisher.

## References

[1] Alicic RZ Rooney MT Tuttle KR. Diabetic Kidney Disease: Challenges, Progress, and Possibilities. Clin J Am Soc Nephrol (2017) 12(12):2032–45. doi: 10.2215/CJN.11491116 PMC5718284 28522654

[2] Harding JL Pavkov ME Magliano DJ Shaw JE Gregg EW. Global Trends in Diabetes Complications: A Review of Current Evidence. Diabetologia (2019) 62(1):3–16. doi: 10.1007/s00125-018-4711-2 30171279

[3] Jin Q Luk AO Lau ES Tam CHT Ozaki R Lim CKP. Nonalbuminuric Diabetic Kidney Disease and Risk of All-Cause Mortality and Cardiovascular and Kidney Outcomes in Type 2 Diabetes: Findings From the Hong Kong Diabetes Biobank. Am J Kidney Dis (2022) S0272-6386(21):01052–0. doi: 10.1053/j.ajkd.2021.11.011 34999159

[4] Wan H Zhang K Wang Y Chen Y Zhang W Xia F. The Associations Between Gonadal Hormones and Serum Uric Acid Levels in Men and Postmenopausal Women With Diabetes. Front Endocrinol (2020) 11:55. doi: 10.3389/fendo.2020.00055 PMC7044188 32153501

[5] Sabanayagam C Chee ML Banu R Cheng CY Lim SC Tai ES. Association of Diabetic Retinopathy and Diabetic Kidney Disease With All-Cause and Cardiovascular Mortality in a Multiethnic Asian Population. JAMA Net Open (2019) 2(3):e191540. doi: 10.1001/jamanetworkopen.2019.1540 PMC6450319 30924904

[6] Labrie F Luu-The V Bélanger A Lin SX Simard J Pelletier G. Is Dehydroepiandrosterone a Hormone? J Endocrinol (2005) 187(2):169–96. doi: 10.1677/joe.1.06264 16293766

[7] Rutkowski K Sowa P Rutkowska-Talipska J Kuryliszyn-Moskal A Rutkowski R. Dehydroepiandrosterone (DHEA): Hypes and Hopes. Drugs (2014) 74(11):1195–207. doi: 10.1007/s40265-014-0259-8 25022952

[8] Altman R Motton DD Kota RS Rutledge JC. Inhibition of Vascular Inflammation by Dehydroepiandrosterone Sulfate in Human Aortic Endothelial Cells: Roles of PPARalpha and NF-kappaB. Vasc Pharmacol (2008) 48(2-3):76–84. doi: 10.1016/j.vph.2007.12.002 PMC3656484 18255343

[9] Liu D Iruthayanathan M Homan LL Wang Y Yang L Wang Y. Dehydroepiandrosterone Stimulates Endothelial Proliferation and Angiogenesis Through Extracellular Signal-Regulated Kinase 1/2-Mediated Mechanisms. Endocrinology (2008) 149(3):889–98. doi: 10.1210/en.2007-1125 PMC2275364 18079198

[10] Bonnet S Paulin R Sutendra G Dromparis P Roy M Watson KO. Dehydroepiandrosterone Reverses Systemic Vascular Remodeling Through the Inhibition of the Akt/GSK3-{Beta}/NFAT Axis. Circulation (2009) 120(13):1231–40. doi: 10.1161/CIRCULATIONAHA.109.848911 19752325

[11] Brahimaj A Muka T Kavousi M Laven JS Dehghan A Franco OH. Serum Dehydroepiandrosterone Levels are Associated With Lower Risk of Type 2 Diabetes: The Rotterdam Study. Diabetologia (2017) 60(1):98–106. doi: 10.1007/s00125-016-4136-8 27771738 PMC6518366

[12] Tivesten Å Vandenput L Carlzon D Nilsson M Karlsson MK Ljunggren Ö. Dehydroepiandrosterone and Its Sulfate Predict the 5-Year Risk of Coronary Heart Disease Events in Elderly Men. J Am Coll Cardiol (2014) 64(17):1801–10. doi: 10.1016/j.jacc.2014.05.076 25443702

[13] Ohlsson C Labrie F Barrett-Connor E Karlsson MK Ljunggren O Vandenput L. Low Serum Levels of Dehydroepiandrosterone Sulfate Predict All-Cause and Cardiovascular Mortality in Elderly Swedish Men. J Clin Endocrinol Metab (2010) 95(9):4406–14. doi: 10.1210/jc.2010-0760 20610590

[14] Veronese N Trevisan C De Rui M Bolzetta F Maggi S Zambon S. Serum Dehydroepiandrosterone Sulfate and Risk for Type 2 Diabetes in Older Men and Women: The Pro. V.A Study Can J Diabetes (2016) 40(2):158–63. doi: 10.1016/j.jcjd.2015.09.013 26923336

[15] Fukui M Kitagawa Y Nakamura N Kadono M Hasegawa G Yoshikawa T. Association Between Urinary Albumin Excretion and Serum Dehydroepiandrosterone Sulfate Concentration in Male Patients With Type 2 Diabetes: A Possible Link Between Urinary Albumin Excretion and Cardiovascular Disease. Diabetes Care (2004) 27(12):2893–7. doi: 10.2337/diacare.27.12.2893 15562203

[16] Tomaszewski M Charchar FJ Maric C Kuzniewicz R Gola M Grzeszczak W. Inverse Associations Between Androgens and Renal Function: The Young Men Cardiovascular Association (YMCA) Study. Am J hypertens (2009) 22(1):100–5. doi: 10.1038/ajh.2008.307 PMC2808108 19096379

[17] Levey AS Stevens LA Schmid CH Zhang YL Castro AF 3rd Feldman HI. A New Equation to Estimate Glomerular Filtration Rate. Ann Internal Med (2009) 150(9):604–12. doi: 10.7326/0003-4819-150-9-200905050-00006 PMC2763564 19414839

[18] Diabetes branch of the Chinese Medical Association. Guidelines for the Prevention and Treatment of Type 2 Diabetes in China (2020 Edition). Chin J Endocrinol Metab (2021) 37(04):311–98. doi: 10.3760/cma.j.cn311282-20210304-00142

[19] The Joint Committee of Chinese Hypertension Prevention Guide. Guidelines on Prevention and Treatment of Hypertension in China (2018 Edition). Chin J Cardiovasc (2019) 24(01):24–56. doi: 10.3969/j.issn.1007-5410.2019.01.002

[20] The Joint Committee of Chinese Adult Dyslipidemia Prevention Guide. Guidelines on Prevention and Treatment of Dyslipidemia in Chinese Adults (2016 Edition). Chin J Cardiol (2016) 44(10):833–53. doi: 10.3760/cma.j.issn.0253-3758.2016.10.005

[21] Tuttle KR Bakris GL Bilous RW Chiang JL de Boer IH Goldstein-Fuchs J. Diabetic Kidney Disease: A Report From an ADA Consensus Conference. Diabetes Care (2014) 37(10):2864–83. doi: 10.2337/dc14-1296 PMC4170131 25249672

[22] Feldman HA Johannes CB Araujo AB Mohr BA Longcope C McKinlay JB. Low Dehydroepiandrosterone and Ischemic Heart Disease in Middle-Aged Men: Prospective Results From the Massachusetts Male Aging Study. Am J Epidemiol (2001) 153(1):79–89. doi: 10.1093/aje/153.1.79 11159150

[23] Wang C Zhang W Wang Y Wan H Chen Y Xia F. Novel Associations Between Sex Hormones and Diabetic Vascular Complications in Men and Postmenopausal Women: A Cross-Sectional Study. Cardiovasc Diabetol (2019) 18(1):97. doi: 10.1186/s12933-019-0901-6 31366359 PMC6668151

[24] Jesse RL Loesser K Eich DM Qian YZ Hess ML Nestler JE. Dehydroepiandrosterone Inhibits Human Platelet Aggregation *In Vitro* and In Vivo. Ann New York Acad Sci (1995) 774:281–90. doi: 10.1111/j.1749-6632.1995.tb17388.x-i1 8597466

[25] Aribas E Ahmadizar F Mutlu U Ikram MK Bos D Laven JSE. Sex Steroids and Markers of Micro- and Macrovascular Damage Among Women and Men From the General Population. Eur J Prev Cardiol (2021) zwaa031. doi: 10.1093/eurjpc/zwaa031 33580786

[26] de Heredia FP Larqué E Zamora S Garaulet M. Dehydroepiandrosterone Modifies Rat Fatty Acid Composition of Serum and Different Adipose Tissue Depots and Lowers Serum Insulin Levels. J Endocrinol (2009) 201(1):67–74. doi: 10.1677/JOE-08-0432 19144736

[27] Sánchez J Pérez-Heredia F Priego T Portillo MP Zamora S Garaulet M. Dehydroepiandrosterone Prevents Age-Associated Alterations, Increasing Insulin Sensitivity. J Nutr Biochem (2008) 19(12):809–18. doi: 10.1016/j.jnutbio.2007.10.005 18482832

[28] Kiersztan A Nagalski A Nalepa P Tempes A Trojan N Usarek M. DHEA-Induced Modulation of Renal Gluconeogenesis, Insulin Sensitivity and Plasma Lipid Profile in the Control- and Dexamethasone-Treated Rabbits. Metab Stud Biochimie (2016) 121:87–101. doi: 10.1016/j.biochi.2015.11.019 26616007

[29] Kiersztan A Gaanga K Witecka A Jagielski AK. DHEA-Pretreatment Attenuates Oxidative Stress in Kidney-Cortex and Liver of Diabetic Rabbits and Delays Development of the Disease. Biochimie (2021) 185:135–45. doi: 10.1016/j.biochi.2021.03.010 33771656

[30] Kiersztan A Trojan N Tempes A Nalepa P Sitek J Winiarska K. DHEA Supplementation to Dexamethasone-Treated Rabbits Alleviates Oxidative Stress in Kidney-Cortex and Attenuates Albuminuria. J Steroid Biochem Mol Biol (2017) 174:17–26. doi: 10.1016/j.jsbmb.2017.07.021 28782595

[31] Aragno M Tamagno E Gatto V Brignardello E Parola S Danni O. Dehydroepiandrosterone Protects Tissues of Streptozotocin-Treated Rats Against Oxidative Stress. Free Radical Biol Med (1999) 26(11-12):1467–74. doi: 10.1016/S0891-5849(99)00012-X 10401610

[32] Aragno M Parola S Brignardello E Manti R Betteto S Tamagno E. Oxidative Stress and Eicosanoids in the Kidneys of Hyperglycemic Rats Treated With Dehydroepiandrosterone. Free Radical Biol Med (2001) 31(8):935–42. doi: 10.1016/S0891-5849(01)00669-4 11595378

[33] Brignardello E Gallo M Aragno M Manti R Tamagno E Danni O. Dehydroepiandrosterone Prevents Lipid Peroxidation and Cell Growth Inhibition Induced by High Glucose Concentration in Cultured Rat Mesangial Cells. J Endocrinol (2000) 166(2):401–6. doi: 10.1677/joe.0.1660401 10927629

[34] Dhatariya K Bigelow ML Nair KS. Effect of Dehydroepiandrosterone Replacement on Insulin Sensitivity and Lipids in Hypoadrenal Women. Diabetes (2005) 54(3):765–9. doi: 10.2337/diabetes.54.3.765 15734854

[35] Weiss EP Villareal DT Fontana L Han DH Holloszy JO. Dehydroepiandrosterone (DHEA) Replacement Decreases Insulin Resistance and Lowers Inflammatory Cytokines in Aging Humans. Aging (2011) 3(5):533–42. doi: 10.18632/aging.100327 PMC3156603 21566261

[36] Villareal DT Holloszy JO. Effect of DHEA on Abdominal Fat and Insulin Action in Elderly Women and Men: A Randomized Controlled Trial. Jama (2004) 292(18):2243–8. doi: 10.1001/jama.292.18.2243 15536111

[37] Basu R Dalla Man C Campioni M Basu A Nair KS Jensen MD. Two Years of Treatment With Dehydroepiandrosterone Does Not Improve Insulin Secretion, Insulin Action, or Postprandial Glucose Turnover in Elderly Men or Women. Diabetes (2007) 56(3):753–66. doi: 10.2337/db06-1504 17327446

[38] Jedrzejuk D Medras M Milewicz A Demissie M. Dehydroepiandrosterone Replacement in Healthy Men With Age-Related Decline of DHEA-S: Effects on Fat Distribution, Insulin Sensitivity and Lipid Metabolism. Aging male (2003) 6(3):151–6. doi: 10.1080/tam.6.3.151.156 14628495

[39] Jankowski CM Gozansky WS Van Pelt RE Wolfe P Schwartz RS Kohrt WM. Oral Dehydroepiandrosterone Replacement in Older Adults: Effects on Central Adiposity, Glucose Metabolism and Blood Lipids. Clin Endocrinol (2011) 75(4):456–63. doi: 10.1111/j.1365-2265.2011.04073.x PMC3166648 21521341

[40] Rice SP Agarwal N Bolusani H Newcombe R Scanlon MF Ludgate M. Effects of Dehydroepiandrosterone Replacement on Vascular Function in Primary and Secondary Adrenal Insufficiency: A Randomized Crossover Trial. J Clin Endocrinol Metab (2009) 94(6):1966–72. doi: 10.1210/jc.2008-2636 19318448

[41] Callies F Fassnacht M van Vlijmen JC Koehler I Huebler D Seibel MJ. Dehydroepiandrosterone Replacement in Women With Adrenal Insufficiency: Effects on Body Composition, Serum Leptin, Bone Turnover, and Exercise Capacity. J Clin Endocrinol Metab (2001) 86(5):1968–72. doi: 10.1210/jcem.86.5.7483 11344193

[42] Yamaguchi Y Zampino M Moaddel R Chen TK Tian Q Ferrucci L. Plasma Metabolites Associated With Chronic Kidney Disease and Renal Function in Adults From the Baltimore Longitudinal Study of Aging. Metabolomics (2021) 17(1):9. doi: 10.1007/s11306-020-01762-3 33428023 PMC9220986

[43] Fukui M Kitagawa Y Kamiuchi K Hasegawa G Yoshikawa T Nakamura N. Low Serum Dehydroepiandrosterone Sulfate Concentration is a Predictor for Deterioration of Urinary Albumin Excretion in Male Patients With Type 2 Diabetes. Diabetes Res Clin Pract (2006) 73(1):47–50. doi: 10.1016/j.diabres.2005.11.006 16413943

[44] Fukui M Ose H Nakayama I Hosoda H Asano M Kadono M. Association Between Urinary Albumin Excretion and Serum Dehydroepiandrosterone Sulfate Concentrations in Women With Type 2 Diabetes. Diabetes Care (2007) 30(7):1886–8. doi: 10.2337/dc06-2325 17363753

[45] Hsu HJ Yen CH Chen CK Hsu KH Hsiao CC Lee CC. Low Plasma DHEA-S Increases Mortality Risk Among Male Hemodialysis Patients. Exp gerontol (2012) 47(12):950–7. doi: 10.1016/j.exger.2012.08.012 22974561

[46] Jesić M Jesić M Sajić S Bogićević D Buljugić S Maglajlić S. The Effect of Metabolic and Hormonal Parameters on Microalbuminuria in Adolescents With Type 1 Diabetes Mellitus. Srpski arhiv za celokupno lekarstvo (2013) 141(5-6):315–9. doi: 10.2298/sarh1306315j 23858799

